# Differential impact of coffee quantity and sweetening on body composition parameters and inflammation

**DOI:** 10.3389/fnut.2025.1673677

**Published:** 2025-09-17

**Authors:** Giuseppe Annunziata, Evelyn Frias-Toral, Francesco Campa, Maria Antonieta Touriz Bonifaz, Ludovica Verde, Martina Galasso, Silvia Savastano, Annamaria Colao, Antonio Paoli, Daniel Simancas-Racines, Giovanna Muscogiuri, Luigi Barrea

**Affiliations:** ^1^Facoltà di Scienze Umane, della Formazione e dello Sport, Università Telematica Pegaso, Naples, Italy; ^2^Escuela de Medicina, Universidad Espíritu Santo, Samborondón, Ecuador; ^3^Division of Research, Texas State University, San Marcos, TX, United States; ^4^Department of Biomedical Sciences, University of Padua, Padua, Italy; ^5^Facultad de Ciencias de la Salud, Universidad Católica de Santiago de Guayaquil, Guayaquil, Ecuador; ^6^Facultad de Ciencias Médicas, Universidad de Guayaquil, Guayaquil, Ecuador; ^7^Department of Public Health, University of Naples Federico II, Naples, Italy; ^8^Unità di Endocrinologia, Diabetologia e Andrologia, Dipartimento di Medicina Clinica e Chirurgia, Università degli Studi di Napoli Federico II, Naples, Italy; ^9^Centro Italiano per la cura e il Benessere del Paziente con Obesità (C.I.B.O), Unità di Endocrinologia, Diabetologia e Andrologia, Dipartimento di Medicina Clinica e Chirurgia, Università degli Studi di Napoli Federico II, Naples, Italy; ^10^Cattedra Unesco "Educazione Alla Salute E Allo Sviluppo Sostenibile", University Federico II, Naples, Italy; ^11^Universidad UTE, Facultad de Ciencias de la Salud Eugenio Espejo, Centro de Investigación en Salud Pública y Epidemiología Clínica (CISPEC), Quito, Ecuador; ^12^Dipartimento di Psicologia e Scienze della Salute, Università Telematica Pegaso, Naples, Italy

**Keywords:** coffee, bioactive compounds, bioelectrical impedance analysis, skeletal muscle mass, phase angle, standardised phase angle, appendicular skeletal muscle mass, hs-C reactive protein

## Abstract

**Background:**

Coffee is the most consumed and popular beverage worldwide. The health benefits of its regular, moderate consumption are well known, and include antioxidant and anti-inflammatory effects, as well as metabolic effects, reducing the risk of obesity and related diseases. The available literature, however, provides no information about the effect of coffee consumption on body composition (BC) and inflammation. The present cross-sectional observational study aims to investigate the effect of coffee consumption on BC and inflammation-related parameters, as well as the possible impact of adding sugar and the frequency of consumption.

**Methods:**

Coffee consumption habits, preference for adding sugar and frequency of daily consumption were assessed in 2,556 adults (1,080 men and 1,476 women). BC was assessed using Bioelectrical Impedance Analysis (BIA), whilst high-sensitivity C-reactive protein (hs-CRP) levels were monitored for inflammatory status.

**Results:**

A total of 1,855 subjects (680 men and 1,175 women) were included in the statistical analysis. Compared to non-consumers, coffee consumers showed lower body mass index (BMI), waist girth (WG), and hs-CRP levels, and higher skeletal muscle mass (SMM), appendicular SMM (ASMM), phase angle (PhA), and standardised PhA (SPA) (*p* < 0.001 for all). The same trend was observed for unsweetened coffee consumers compared to subjects consuming sweetened coffee. With increasing coffee consumption, BMI, WG, and hs-CRP generally decreased, whilst SMM and ASMM showed a bell-shaped trend with peak values in those consuming 2–3 cups per day. Similarly, PhA and SPA values were highest among moderate coffee consumers.

**Conclusion:**

These findings suggest that moderate coffee consumption, particularly unsweetened coffee, is associated with more favourable body composition and inflammatory profiles. Given the observational design, causality cannot be established. Nevertheless, the results may inform dietary guidance aimed at supporting muscle maintenance and mitigating obesity-related metabolic risk.

## 1 Introduction

The current literature indicates an effective association between coffee consumption and various health outcomes, including those related to weight and body composition (BC) (1). In this sense, most of the evidence shows that coffee intake is associated with reduced values or reductions in total and abdominal fat mass (FM) (2–5), with the exception of one study reporting opposite data in kidney transplant recipients (6).

The protective effects of coffee consumption would appear to be due to its particular composition, which is characterised by the presence of a number of bioactive compounds, including both alkaloids (such as caffeine) and polyphenols (the main one being chlorogenic acid) (1). These bioactive compounds are responsible for most of the antioxidant and anti-inflammatory effects of coffee, acting through different mechanisms. For caffeine, the mechanisms include the regulation of reactive oxygen species production, the reduction of pro-inflammatory cytokine levels, and the increase of Nrf-2 expression. For chlorogenic acid, they include free radical scavenging, downregulation of pro-inflammatory cytokine gene expression, and regulation of NF-κB activation (1). Similarly, both compounds show metabolic effects: Caffeine activates the sympathetic system, increasing basal metabolic rate, thermogenesis, and lipolysis, and chlorogenic acid downregulates adipogenesis-related genes and upregulates those involved in fatty acid *β*-oxidation. Taken together, these effects result in a general reduction in the risk of metabolic alterations and abdominal obesity (1), highlighting the important nutraceutical potential of coffee (7).

It is important to take into account, however, that the relative amount of these bioactive compounds can vary significantly depending on different aspects, such as the variety of the plant or the different ways in which they are produced (8–10). Similarly, the daily amount of coffee consumed varies considerably among individuals, possibly leading to differences in health effects (11), suggesting related possible differences in the effects on health. A further aspect to consider is that, although promising, the majority of studies that have investigated the associations between coffee consumption and BC have focused on the effects of the beverage on FM levels and distribution, neglecting other important aspects related to both BC and nutritional status, such as fat-free mass (FFM), skeletal muscle mass (SMM), and inflammatory indices (e.g., serum markers of inflammation or surrogate markers). The study of the impact of diet on these BC and nutritional status components is essential for the management of pathological conditions, including obesity-related muscle loss. To the best of our knowledge, only a limited number of human studies (four on Asian and one on American middle-aged subjects) investigated the effect of coffee on muscle, evaluated with different methods, and described inverse associations between this beverage consumption and prevalence of low muscle mass (12–16). In particular, since their main aim was to evaluate the protective effect of coffee against muscle loss, and due to the population analysed, these studies focused only on the assessment of SMM and appendicular SMM (ASMM) indices, normalised by the squared height (12–16).

A further limitation in the literature is the lack of differentiation between coffee consumers who add sugar and those who do not. This distinction is relevant because sweetened coffee, especially when consumed in large amounts, can significantly affect caloric intake and contribute to long-term changes in BC. Chronic sugar intake negatively impacts muscle mass (17) and strength (18), also outlining the mechanisms of action that see the establishment of IR and inflammation (19, 20).

In addition to SMM and ASMM, the assessment of inflammatory status is also complex, given the need to monitor blood parameters. For this reason, it is currently accepted to monitor rapid and non-invasive surrogate markers, including the bioelectrical impedance analysis (BIA) parameter phase angle (PhA), whose prognostic and predictive value of the inflammatory state has been demonstrated (21).

In light of existing knowledge gaps, this cross-sectional observational study aims to examine the association between coffee consumption and various parameters of BC and inflammation in an adult population. In addition, the study will explore how different coffee consumption patterns, particularly regarding sugar use and quantity consumed, may influence these outcomes. This comprehensive approach seeks to clarify the broader nutraceutical potential of coffee and its role in modulating metabolic health and inflammation-related BC markers and may also provide novel insight into the nutritional management of sarcopenic obesity.

## 2 Materials and methods

### 2.1 Design and setting

This is a cross-sectional observational study carried out at the *Centro Italiano per la cura e il Benessere del paziente con Obesità* (C.I.B.O.) of the University Hospital Federico II of Naples, Italy. Subjects were recruited at the outpatient clinic and within the OPERA project (22) from December 2011 to February 2021. The study was conducted in accordance with the Declaration of Helsinki guidelines for human experimentation. The study protocol was approved by the Ethics Committee of the Federico II University Medical School of Naples (n. 239/11). Study purpose and protocol were clearly presented and explained to participants, who provided their written consent. The study was conducted without support from food and/or pharmaceutical companies.

### 2.2 Population study

**Figure 1:**
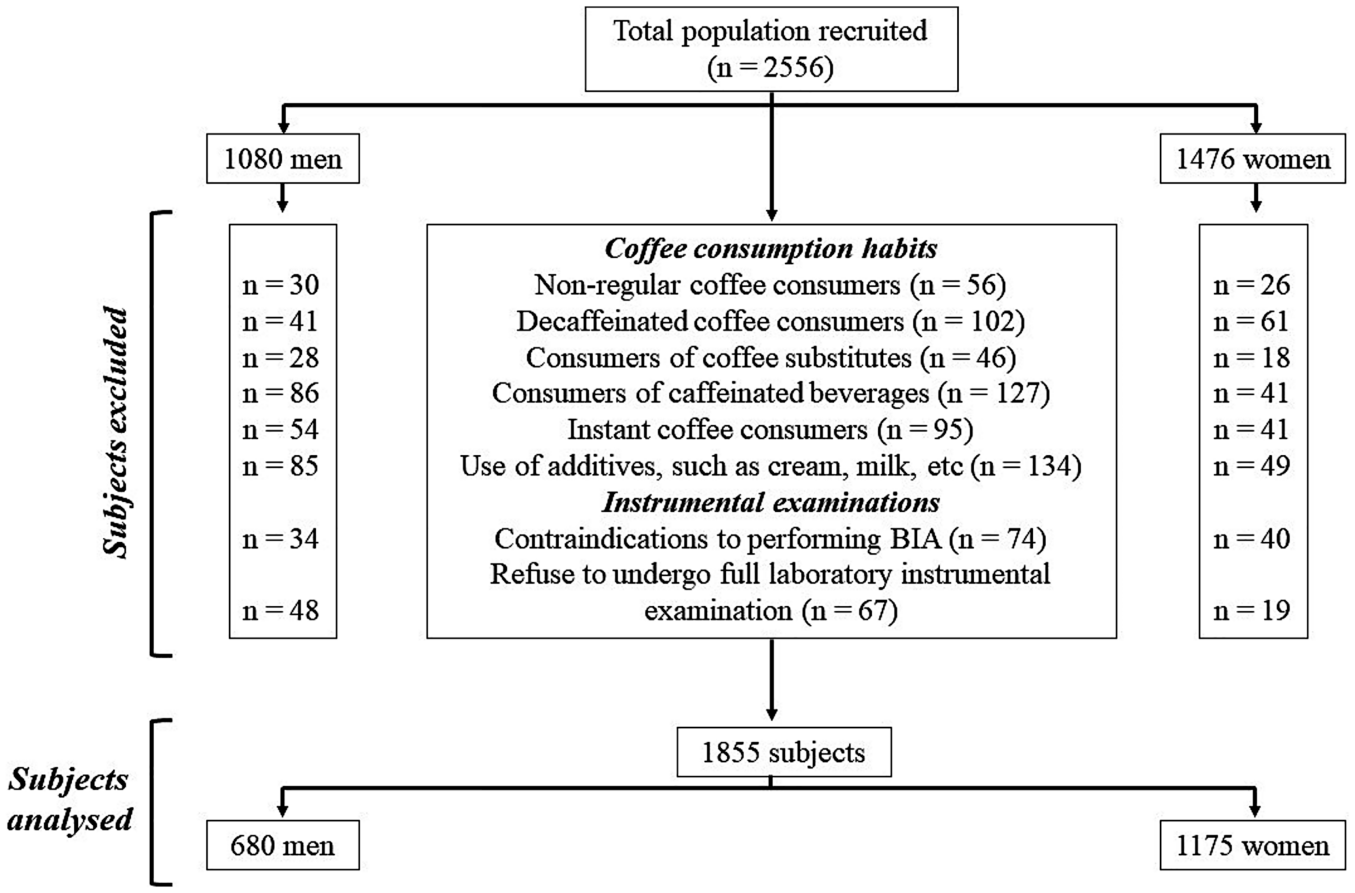
Flowchart of the study.

In this study, we enrolled a total of 2,556 Italian adults (1,080 men and 1,476 women) aged 18–59 years, with a body mass index (BMI) ranging from 19.5 to 69.4 kg/m^2^. All participants were administered an anamnestic questionnaire, from which, in addition to collecting information on any diagnoses and/or pharmacological therapies followed, it was noted that none had any clinical conditions that could alter the fluid balance. In general, all participants were healthy. All women participants were not breastfeeding and were not pregnant; those of childbearing age were assessed during the follicular phase of the menstrual cycle. For selecting study participants, the inclusion criteria were as follows: both sexes aged 18 years or over with a BMI ≥ 18.5 kg/m^2^ and either regular coffee consumers (consuming coffee every day) or non-consumers; among the regular coffee consumers, only subjects consuming caffeinated coffee were included. The following exclusion criteria were as follows: BMI < 18.5 kg/m^2^, diagnosis of chronic diseases (i.e., type 1 and 2 diabetes, mental disorders, cancer, anaemia, renal or liver insufficiency, chronic inflammatory diseases, or endocrine disorders), use of drugs influencing body weight (BW) or fluid balance, vitamins/mineral or antioxidant supplementation, alcohol abuse, specific dietary patterns followed (i.e., ketogenic diet, hypocaloric diet, or vegetarian diet), contraindication to performing BIA (i.e., presence of metal prostheses or electronic medical implants or skin damage), and participation to other studies. With regard to coffee consumption habits, subjects were excluded if they (i) consumed coffee occasionally (not every day), (ii) consumed decaffeinated coffee, (iii) consumed coffee substitutes (e.g., barley coffee and ginseng), (iv) consumed caffeinated beverages (e.g., cola and energy drinks), (v) consumed instant coffee, and (vi) used additives (e.g., cream and milk), as shown in Figure 1.

### 2.3 Coffee consumption assessment

The coffee consumption data were collected using a 7-day food diary record carried out during a face-to-face interview between the subject and a certified nutritionist. In detail, all participants prospectively completed the 7-day food diary record after the nutritionists had provided detailed information on how to fill it out. During the interview, each participant was asked about the amount of coffee consumed (expressed as the number of cups *per* day) and whether sugar was added. As previously reported, one cup of coffee was considered to contain 50 mL of espresso (2). For sugar addition, the average intake of 5 g/cup (corresponding to one sachet or one teaspoon of sugar) was considered. The coffee consumption was analysed in three separate models:

1. Coffee consumption (consumers vs. non-consumers),
2. Daily servings of coffee (1 cup coffee *per* day, 2 cups coffee *per* day, 3 cups coffee *per* day, 4 or more cups of coffee *per* day),
3. Unsweetened coffee vs. sweetened coffee.

### 2.4 Anthropometric measurements

As previously described (23–26), an experienced nutritionist took anthropometric measurements in the morning (between 8 a.m. and 10 a.m.). The subjects, who had been fasting overnight, were asked to remove their shoes and wear only light clothing. BW was measured using a calibrated scale (Seca 711; Seca, Hamburg, Germany) to the nearest 0.1 kg, whilst height was measured using a wall altimeter (Seca 711; Seca, Hamburg, Germany) to the nearest 0.5 cm. The BMI, calculated using the formula [weight (kg)/height^2^ (m^2^)] = BMI (kg/m^2^), was used to classify the study participants as normal weight (18.5–24.9 kg/m^2^), overweight (25.0–29.9 kg/m^2^), grade I obesity (30.0–34.9 kg/m^2^), grade II obesity (35.0–39.9 kg/m^2^), and grade III obesity (>40.0 kg/m^2^) according to the World Health Organisation classification (27). A non-elastic tape with an accuracy of 0.1 cm was used to measure waist girth (WG). The detection was performed on standing subjects, who were asked to put their feet together, keep their arms at their sides, and breathe normally (28). The WG measurement was taken at the midpoint between the iliac crest and the last rib. In subjects with a high degree of adiposity that made it difficult to identify the correct point of reference, the measurement was taken with 0.1 cm at the umbilical level (28). The following WG cutoffs were used: ≥102 and 88 cm for men and women, respectively (29).

### 2.5 Bioelectrical impedance analysis

BIA was performed with a 50 kHz frequency, 800A current analyser (BIA 101 BIVA, Akern Ltd, Pisa, Italy), following the European Society of Parenteral and Enteral Nutrition (ESPEN) Guidelines (30). The device was checked through resistors and capacitors of known value before analysis. The test was conducted on fasting subjects who were asked not to practise heavy exercise for at least 6 h and not drink alcohol in the 24 h before the visit. Subjects were supine with both upper and lower limbs separated from the body. Electrodes (BIATRODES Akern Srl; Florence, Italy) were placed on the skin cleaned with alcohol on the back of the right hand and foot. On the hand, the two electrodes were placed at the phalangeal-metacarpal joint and at the midpoint between the distal projection of the radius and ulna. On the foot, the two electrodes were placed at the transverse arch and at the midpoint between the medial and lateral malleoli. Using BIA, resistance (Rz) and reactance (Xc) were directly measured. From Rx and Xc, PhA was directly calculated using the formula PhA (°) = arctangent of the Xc/R x 180/*π*. Since PhA is strongly influenced by sex, age, and BMI (31), studies have suggested that evaluating the standardised PhA (SPA) may be more accurate (32). SPA, defined as the PhA normalised by sex and age (32), is calculated according to the formula: SPA = [(PhA_obs_ − PhA_ref_)/SD_PhA-ref_], where PhA_obs_ is the value of PhA measured, and PhA_ref_ and SD_PhA-ref_ refer, respectively, to the mean PhA and standard deviation from a reference population stratified by age and sex (33). As BC-related parameters, we evaluated SMM and ASMM. SMM, expressed in kg, was calculated by the Janssen formula: SMM = (*h^2^/*R × 0.401) + (*sex* × 3.825) + (*age* × −0.071) + 5.102, where *h* refers to the height expressed in cm, and constants for sex are 1 for male and 0 for female (34). ASMM, expressed in kg, was calculated using appropriate validated predictive equations incorporating Rz, Xc, sex, age, and weight (35). All BIA parameters were calculated using the Bodygram Plus software (Akern, Florence, Italy).

### 2.6 Measurement of high-sensitivity C-reactive protein (hs-CRP) levels

High-sensitivity C-reactive protein (hs-CRP) levels were assessed on venous blood taken in the morning (between 8:00 and 10:00), after an overnight fast (at least 8 h). The measurement was performed using a high-sensitivity nephelometric test (CardioPhase hsCRP kit, Siemens Healthcare Diagnostics, Marburg, Germany), with a minimum detection limit = 0.01 mg/L and intra- and inter-test variability of <7%. As indicated by the Centres for Disease Control and Prevention and American Heart Association guidelines, based on hs-CRP levels, study participants were classified into three cardiovascular risk categories: low (<1.0 mg/L), intermediate (1.0–3.0 mg/L), and high (≥3.0 mg/L) (36).

### 2.7 Physical activity and smoking habits

Physical activity levels (PALs) and cigarette smoking habits were assessed in all participants by administering a standardised questionnaire, as previously reported in other studies (23, 26, 37). Participants were considered active if they practised at least 30 min of aerobic physical activity *per* day. Similarly, participants were considered to be current smokers if they smoked at least one cigarette *per* day, as we have previously reported (25, 38).

### 2.8 Statistical analysis

The statistical analysis was conducted using the IBM SPSS Statistics Software and MedCalc® package (PASW Version 21.0, SPSS Inc., Chicago, IL, USA, and version 12.3.0, 1993–2012 MedCalc Software bvba, MedCalc Software, Mariakerke, Belgium, respectively). Graphs were created with GraphPad Prism v9.1.1. Data are expressed as mean ± standard deviation (SD). Differences between two groups (coffee consumption: YES/NO and coffee consumption: unsweetened/sweetened) were analysed using an unpaired *t*-test for independent samples. When more than two groups were compared (number of cups of coffee consumed, 0–4), analysis of variance (ANOVA) tests were performed, followed by Bonferroni *post-hoc* analysis. A chi-square (χ2) test was used to assess the significance of differences in frequency distribution (sex, BMI, WG, hs-CRP, PAL smoking, and number of cups). Proportional odds ratio (OR) models, along with 95% confidence interval (CI) and R^2^, were used to assess the association between quantitative variables (coffee consumption: YES/NO and coffee consumption: unsweetened/sweetened) and study parameters.

## 3 Results

In this study, a total of 1,855 Italian men and women (680 and 1,175, respectively), aged 18–59 years, with a body mass index (BMI) ranging from 19.5 to 69.4 kg/m^2^, were analysed.

**Figure 2:**
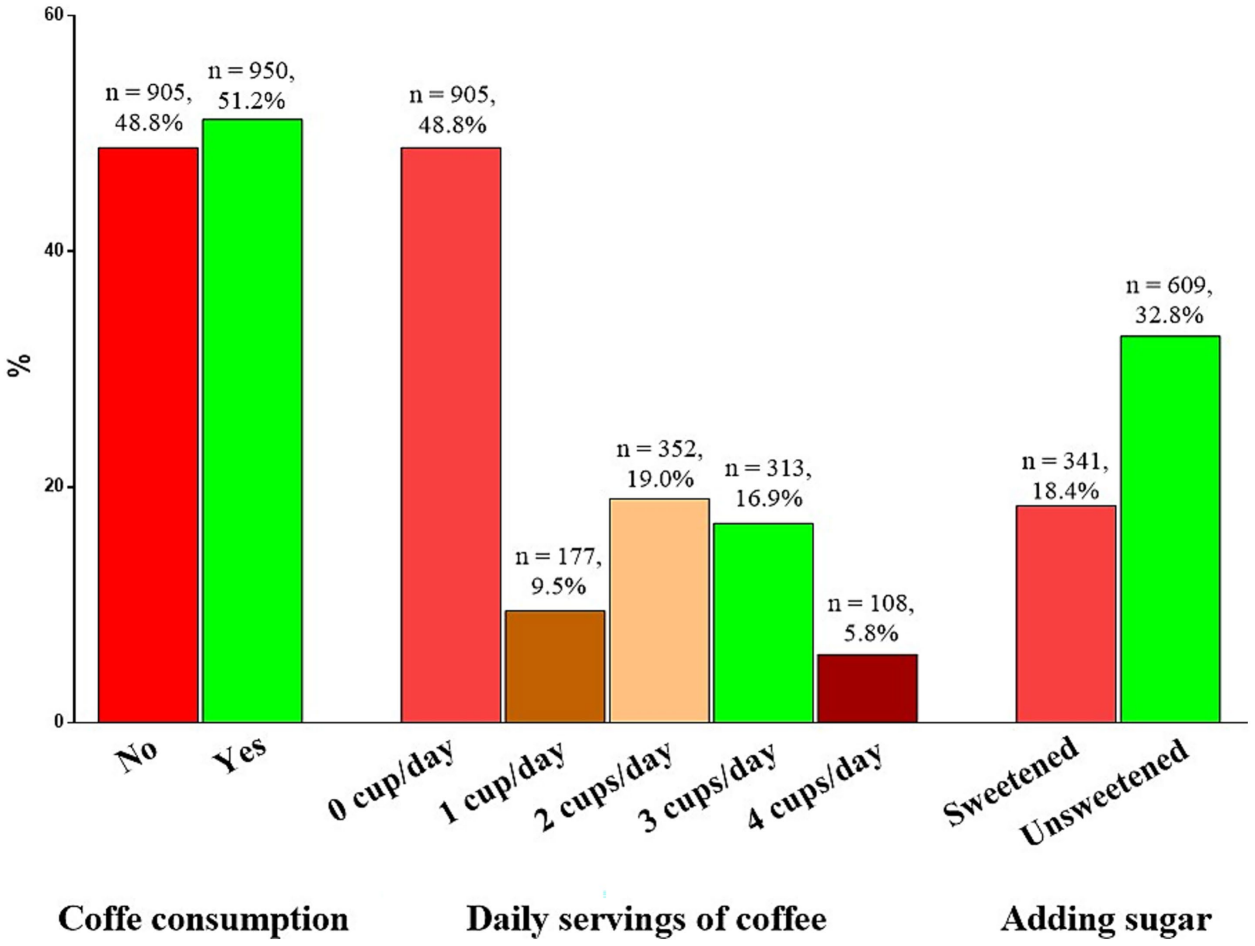
Coffee consumption habits among the study population.

The coffee consumption is shown in Figure 2. In particular, most of the participants consumed coffee (950 subjects, 51.2%). Considering the daily servings of coffee consumption, the majority of participants consumed 2 cups of coffee *per* day (352 subjects, 19.0%). Regarding the addition of sugar to coffee, the majority of participants reported consuming unsweetened coffee (308 subjects, 32.8%).

The total study population (1,855 subjects, 680 men and 1,175 women) was stratified by coffee consumption habit. A total of 950 subjects were habitual coffee consumers (51.21%), whilst 905 subjects did not consume coffee (48.79%). As shown in Table 1, although no differences in average age were reported (*p* = 0.170), coffee consumers had lower BMI (*Δ* = −10.58 kg/m^2^, *p* < 0.001), WG (Δ = −23.33 cm, *p* < 0.001), and hs-CRP levels (Δ = −1.13 mg/L, *p* < 0.001) than non-consumers. On the other hand, coffee consumers had higher SMM (Δ = +4.84 kg, *p* < 0.001), ASMM (Δ = +0.67 kg, *p* < 0.001), PhA (Δ = +0.83°, *p* < 0.001), and SPA (Δ = +0.92, *p* < 0.001) than non-consumers.

**Table 1:** Age, anthropometric and body composition parameters, and inflammatory biomarkers of study participants stratified by coffee consumption.

Parameters	Coffee consumption (YES [*n* = 950]/NO [*n =* 905])	Mean±SD	*p*-value
Age (years)	No	34.91 ± 11.46	0.170
	Yes	34.19 ± 11.00	
BMI (kg/m^2^)	No	40.33 ± 6.82	**<0.001**
	Yes	29.75 ± 6.41	
WG (cm)	No	116.77 ± 23.33	**<0.001**
	Yes	93.44 ± 20.05	
Rz (Ohm)	No	504.16 ± 87.86	**<0.001**
	Yes	452.41 ± 79.50	
Xc (Ohm)	No	46.91 ± 10.31	**<0.001**
	Yes	48.64 ± 9.43	
PhA (°)	No	5.32 ± 0.72	**<0.001**
	Yes	6.15 ± 0.69	
SPA (°)	No	−0.41 ± 0.95	**<0.001**
	Yes	0.51 ± 1.30	
SMM (kg)	No	26.22 ± 5.22	**<0.001**
	Yes	31.06 ± 6.75	
ASMM (kg)	No	24.32 ± 4.30	**<0.001**
	Yes	24.99 ± 5.19	
hs-CRP (mg/L)	No	3.31 ± 3.16	**<0.001**
	Yes	2.18 ± 1.67	

Data are expressed as mean ± standard deviation. A *p*-value in bold type denotes a significant difference (*p* < 0.05). SD, standard deviation; BMI, body mass index; WG, waist girth; Rz, resistance; Xc, reactance; PhA, phase angle; SPA, standardised phase angle; SMM, skeletal muscle mass; ASMM, appendicular skeletal muscle mass; hs-CRP, high-sensitive C-reactive protein.

As can be seen in Table 2, a higher percentage of men reported being coffee consumers than women (21.70 *vs.* 78.30%, *p* < 0.001). Interesting differences were observed with regard to BMI categories. Among non-coffee drinkers, an increase in the percentage of non-coffee consumption was observed as the BMI category increased. Similarly, a higher percentage of non-coffee consumers had WG values above the cutoff (88.00 *vs.* 39.80%, *p* < 0.001) than coffee consumers. Regarding inflammatory status, non-coffee consumers had higher cardiovascular risk percentages than coffee consumers (*p* < 0.001 for all risk categories). Finally, regarding lifestyle habits, non-coffee consumers were more physically active and smoked less than coffee consumers (*p* < 0.001 and *p* = 0.003, respectively).

**Table 2:** Frequency distribution of coffee consumers and non-consumers among sex, anthropometric and cardiovascular risk categories, PAL, and smoking habit.

Categories	Coffee consumption				χ^2^	*p*-value
	NO (*n =* 905)		YES (*n =* 950)			
	*N*	%	*N*	%		
Sex						
Men	196	21.70	484	50.90	169.98	**<0.001**
Women	709	78.30	466	49.10		
BMI						
Normal-weight	10	1.10	271	28.50	269.00	**<0.001**
Overweight	49	5.40	273	28.70	174.12	**<0.001**
I grade obesity	122	13.50	207	21.80	21.36	**<0.001**
II grade obesity	271	29.90	134	14.10	67.21	**<0.001**
III grade obesity	453	50.10	65	6.80	427.87	**<0.001**
WG						
<Cutoff	109	12.00	572	60.20	460.72	**<0.001**
>Cutoff	769	88.00	378	39.80		
hs-CRP						
<1.0 mg/L	126	13.90	190	20.00	11.69	**<0.001**
1.0–3.0 mg/L	413	45.60	548	57.70	26.47	**<0.001**
>3.0 mg/L	366	40.40	212	22.30	70.15	**<0.001**
PAL						
Yes	651	71.90	540	56.80	45.28	**<0.001**
No	254	28.10	410	43.20		
Smoking						
Yes	645	71.30	726	76.40	6.11	**0.013**
No	260	28.70	224	23.60		

Data are expressed as number (*N*) and percentage (%). A *p*-value in bold type denotes a significant difference (*p* < 0.05). BMI, Body mass index; WG, waist girth; hs-CRP, high-sensitive C-reactive protein; PAL, physical activity level.

Table 3 reports the differences in age, anthropometric measurements, and BC parameters, and inflammatory biomarkers of the study participants stratified according to the consumers of sweetened coffee vs. unsweetened coffee. Except for age, Xc, and ASMM, all other parameters evaluated in the study showed statistically significant differences between the two groups. In detail, BMI (*Δ* = −8.09 kg/m^2^, *p* < 0.001) and WG (Δ = −21.51 cm, *p* < 0.001) measurements were lower in consumers of unsweetened coffee compared to consumers of sweetened coffee, as well as hs-CRP levels (Δ = −1.25 mg/L, *p* < 0.001). On the contrary, PhA (Δ = +0.53°, *p* < 0.001), SPA (Δ = +0.75°, *p* < 0.001), and SMM (Δ = +2.85 kg, *p* < 0.001) were higher in consumers of unsweetened coffee than in those who consumed coffee with sugar (Table 3).

**Table 3:** Age, anthropometric and body composition parameters, and inflammatory biomarkers of study participants stratified by consumers of sweetened coffee vs. unsweetened coffee.

Parameters	Coffee consumption (unsweetened [*n* = 609]/sweetened [*n =* 341])	Mean±SD	*p*-value
Age (years)	Unsweetened	34.22 ± 10.86	0.930
	Sweetened	34.15 ± 11.28	
BMI (kg/m^2^)	Unsweetened	26.84 ± 4.40	**<0.001**
	Sweetened	34.93 ± 6.16	
WG (cm)	Unsweetened	84.44 ± 15.58	**<0.001**
	Sweetened	105.95 ± 21.05	
Rz (Ohm)	Unsweetened	439.33 ± 73.79	**<0.001**
	Sweetened	475.75 ± 83.96	
Xc (Ohm)	Unsweetened	48.68 ± 8.69	0.877
	Sweetened	48.58 ± 10.64	
PhA (°)	Unsweetened	6.34 ± 0.61	**<0.001**
	Sweetened	5.81 ± 0.63	
SPA (°)	Unsweetened	0.78 ± 1.28	**<0.001**
	Sweetened	0.03 ± 1.20	
SMM (kg)	Unsweetened	32.09 ± 7.00	**<0.001**
	Sweetened	29.24 ± 5.85	
ASMM (kg)	Unsweetened	24.86 ± 5.30	0.488
	Sweetened	25.10 ± 4.93	
hs-CRP (mg/L)	Unsweetened	1.73 ± 1.17	**<0.001**
	Sweetened	2.98 ± 2.09	

Data are expressed as mean ± standard deviation. A *p*-value in bold type denotes a significant difference (*p* < 0.05). SD, standard deviation; BMI, body mass index; WG, waist girth; Rz, resistance; Xc, reactance; PhA, phase angle; SPA, standardised phase angle; SMM, skeletal muscle mass; ASMM, appendicular skeletal muscle mass; hs-CRP, high-sensitive C-reactive protein.

Table 4 shows the frequency distribution of consumers of sweetened coffee compared to unsweetened coffee according to sex, anthropometric and cardiovascular risk categories, PAL, and smoking habits. Among the unsweetened coffee consumers, there was a higher percentage of men than women (54.20% vs. 45.80%, respectively); the percentage progressively decreased across BMI categories, from normal weight (42.20%) to grade III obesity (0.30%), with the highest prevalence of WG values below the cutoff (78.20%). With regard to inflammatory status, the highest percentage of unsweetened coffee consumers had hs-CRP values between 1.0 and 3.0 mg/L (65.20%). Regarding lifestyle habits, the unsweetened coffee consumers were physically active (51.10%), whilst no differences in cigarette smoking habits were observed (*p* = 0.258). Finally, among unsweetened coffee consumers, the largest proportion consumed 3 cups *per* day (38.10%), whilst among sweetened coffee consumers, the largest proportion consumed 2 cups *per* day (35.80%); Table 4.

**Table 4:** Frequency distribution of consumers of sweetened coffee vs. unsweetened coffee among sex, anthropometric and cardiovascular risk categories, PAL, and smoking habit.

Categories	Coffee consumption				χ^2^	*p*-value
	Unsweetened (*n =* 609)		Sweetened (*n =* 341)			
	*N*	%	*N*	%		
Sex						
Men	330	54.20	154	45.20	6.77	**0.009**
Women	279	45.80	187	54.80		
BMI						
Normal-weight	257	42.20	14	4.10	153.73	**<0.001**
Overweight	216	35.50	57	16.70	36.63	**<0.001**
I grade obesity	100	16.40	107	31.40	27.83	**<0.001**
II grade obesity	34	5.60	100	29.30	99.76	**<0.001**
III grade obesity	2	0.30	63	18.50	110.11	**<0.001**
WG						
<Cutoff	476	78.20	96	28.20	226.11	**<0.001**
>Cutoff	133	21.80	245	71.80		
hs-CRP						
<1.0 mg/L	150	24.60	40	11.70	21.94	**<0.001**
1.0–3.0 mg/L	397	65.20	151	44.30	39.29	**<0.001**
>3.0 mg/L	62	10.20	150	44.00	142.18	**<0.001**
PAL						
Yes	331	51.10	229	67.20	22.41	**<0.001**
No	298	48.90	112	32.80		
Smoking						
Yes	473	77.70	253	74.20	1.28	0.258
No	136	22.30	88	25.80		
Number of cups						
1	83	13.60	94	27.60	27.60	**<0.001**
2	230	37.80	122	35.80	1.27	0.261
3	232	38.10	81	23.80	19.71	**<0.001**
4	64	10.50	44	12.90	1.02	0.313

Data are expressed as number (*N*) and percentage (%). A *p*-value in bold type denotes a significant difference (*p* < 0.05). BMI, body mass index; WG, waist girth; hs-CRP, high-sensitive C-reactive protein; PAL, physical activity level.

In Table 5, differences in age, anthropometric parameters, BC, and inflammatory biomarkers are shown for study participants stratified by the number of cups of coffee consumed *per* day. Although no differences in average age were reported (*p* = 0.257), a J-shaped trend was observed for BMI, WG, and hs-CRP (*p* < 0.001 for all). For these parameters, values tended to decrease as the number of cups consumed increased from 0 to 3 and then increased again in subjects consuming ≥4 cups daily. On the other hand, a different trend was observed for SMM and ASMM. For all these parameters, the relative values followed a bell-shaped trend from 0 to 3 cups of coffee consumed and then increased again in subjects consuming ≥4 cups daily. A complete bell-shaped curve was observed for PhA and SPA values, with the highest found in subjects consuming 2 and 3 coffee cups, respectively (Table 5).

**Table 5:** Age, anthropometric, body composition parameters, and inflammatory biomarkers of study participants stratified by the number of cups of coffee consumed.

Parameters	Cups of coffee consumed	Mean±SD	*p*-value
Age (years)	0 (*n* = 905)	34.91 ± 11.46^a^	0.257
	1 (*n* = 177)	34.25 ± 10.98^a^	
	2 (*n* = 352)	34.72 ± 10.61^a^	
	3 (*n* = 313)	33.28 ± 11.09^a^	
	≥4 (*n* = 108)	35.04 ± 12.00^a^	
BMI (kg/m^2^)	0 (*n* = 905)	40.33 ± 6.82^a^	**<0.001**
	1 (*n* = 177)	34.71 ± 6.86^b^	
	2 (*n* = 352)	30.37 ± 5.91^c^	
	3 (*n* = 313)	26.03 ± 6.41^d^	
	≥4 (*n* = 108)	30.33 ± 5.27^b^	
WG (cm)	0 (*n* = 905)	116.77 ± 23.33^a^	**<0.001**
	1 (*n* = 177)	107.99 ± 21.01^b^	
	2 (*n* = 352)	96.06 ± 19.10^c^	
	3 (*n* = 313)	80.96 ± 14.50^d^	
	≥4 (*n* = 108)	97.25 ± 13.64^b^	
Rz (Ohm)	0 (*n* = 905)	504.16 ± 87.86^a^	**<0.001**
	1 (*n* = 177)	469.03 ± 79.56^b^	
	2 (*n* = 352)	451.24 ± 74.51^b^	
	3 (*n* = 313)	454.04 ± 82.49^b^	
	≥4 (*n* = 108)	424.21 ± 79.39^c^	
Xc (Ohm)	0 (*n* = 905)	46.91 ± 10.31^a,c^	**<0.001**
	1 (*n* = 177)	50.31 ± 11.23^b^	
	2 (*n* = 352)	49.65 ± 9.04^b^	
	3 (*n* = 313)	48.09 ± 8.50^a,b^	
	≥4 (*n* = 108)	44.24 ± 8.65^c^	
PhA (°)	0 (*n* = 905)	5.32 ± 0.72^a^	**<0.001**
	1 (*n* = 177)	6.12 ± 0.86^b,c,d^	
	2 (*n* = 352)	6.30 ± 0.69^b^	
	3 (*n* = 313)	6.07 ± 0.50^c^	
	≥4 (*n* = 108)	5.97 ± 0.58^c,d^	
SPA (°)	0 (*n* = 905)	−0.41 ± 0.95^a^	**<0.001**
	1 (*n* = 177)	0.10 ± 1.11^b^	
	2 (*n* = 352)	0.48 ± 1.29^c^	
	3 (*n* = 313)	1.07 ± 1.27^d^	
	≥4 (*n* = 108)	−0.34 ± 0.93^a^	
SMM (kg)	0 (*n* = 905)	26.22 ± 5.22^a^	**<0.001**
	1 (*n* = 177)	30.67 ± 5.76^b^	
	2 (*n* = 352)	31.74 ± 6.57^b,c^	
	3 (*n* = 313)	29.18 ± 6.88^b,d^	
	≥4 (*n* = 108)	37.97 ± 6.54^e^	
ASMM (kg)	0 (*n* = 905)	24.32 ± 4.29^a^	**<0.001**
	1 (*n* = 177)	26.20 ± 4.55^b,d^	
	2 (*n* = 352)	25.68 ± 5.06^b^	
	3 (*n* = 313)	22.51 ± 4.95^c^	
	≥4 (*n* = 108)	27.58 ± 4.33^d^	
hs-CRP (mg/L)	0 (*n* = 905)	3.31 ± 3.16^a^	**<0.001**
	1 (*n* = 177)	3.38 ± 2.37^a^	
	2 (*n* = 352)	2.10 ± 0.99^b^	
	3 (*n* = 313)	0.99 ± 0.64^c^	
	≥4 (*n* = 108)	3.89 ± 1.26^a^	

Data are expressed as mean ± standard deviation. A *p*-value in bold type denotes a significant difference (*p* < 0.05). SD, standard deviation; BMI, body mass index; WG, waist girth; Rz, resistance; Xc, reactance; PhA, phase angle; SPA, standardised phase angle; SMM, skeletal muscle mass; ASMM, appendicular skeletal muscle mass; hs-CRP, high-sensitive C-reactive protein.

Table 6 shows the frequency distribution of the study parameters according to the number of cups of coffee consumed *per* day. Among the study participants, the highest number of women did not consume coffee regularly (*n* = 709), whilst the lowest consumed ≥4 cups daily (*n* = 22); on the other hand, the highest number of men consumed 2 cups daily (*n* = 215), whilst the lowest consumed 3 cups daily (*n* = 78). According to the BMI categories: (i) the highest number of subjects with normal weight consumed 3 cups daily (*n* = 167), whilst the lowest consumed 0 or 1 cup (*n* = 10 in both groups), (ii) the highest number of subjects with overweight consumed 2 cups daily (*n* = 119), whilst the lowest consumed ≥4 cups daily (*n* = 22), (iii) the highest number of subjects with grade I obesity did not consume coffee (*n* = 122), whilst the lowest consumed ≥4 cups daily (*n* = 29), (iv) the highest number of subjects with grade II obesity did not consume coffee (*n* = 271), whilst the lowest consumed 3 cups daily (*n* = 14), and (v) the highest number of subjects with grade III obesity did not consume coffee (*n* = 453), whilst the lowest consumed 3 or ≥4 cups daily (*n* = 2 in both groups). Regarding the WG, for 0 and 1 cup consumed, the highest prevalence of subjects had values above the cutoff (88.00 and 66.70%, respectively), whilst for 2, 3, and ≥4 cups consumed, the highest prevalence of subjects had values below the cutoff (57.10, 81.20, and 53.70%, respectively). With regard to the inflammatory status, for 0, 1, and 2 cups consumed, the highest prevalence of subjects had values of hs-CRP ranging 1.0–3.0 mg/L (45.60, 58.80, and 78.70%, respectively); on the other hand, for 3 cups consumed, the highest prevalence of subjects had values of hs-CRP < 1.0 mg/L (54.30%), whilst for ≥4 cups consumed, the highest prevalence of subjects had values of hs-CRP > 3.0 mg/L (76.90%). Regarding PAL, the highest prevalence of subjects consuming 0, 1, 2, and ≥4 cups was active (71.90, 72.90, 58.00, and 68.50%, respectively), whilst the highest prevalence of subjects consuming 3 cups was inactive (55.00%). The majority of study participants were current smokers, regardless of the consumption of coffee. Finally, the highest prevalence of subjects consuming 1 cup preferred sweetened coffee (53.10%), whilst the highest prevalence of subjects consuming 2, 3, and ≥4 cups preferred unsweetened coffee (65.30, 74.10, and 59.30%, respectively) (Table 6).

**Table 6:** Frequency distribution of study participants among sex, anthropometric and cardiovascular risk categories, PAL, and smoking habit according to the number of cups of coffee consumed.

Categories	Number of cups of coffee consumed				
	0 (*n =* 905)	1 (*n* = 177)	2 (*n =* 352)	3 (*n =* 313)	≥4 (*n =* 108)
Sex (*N*, %)					
Men	196, 21.70	105, 59.30	215, 61.10	78, 24.90	86, 79.6
Women	709, 8.30	72, 40.70	137, 38.90	235, 75.10	22, 20.40
BMI (*N*, %)					
Normal-weight	10, 1.10	10, 5.60	72, 20.50	167, 53.40	22, 20.40
Overweight	49, 5.40	45, 25.40	119, 33.80	80, 25.60	29, 26.90
I grade obesity	122, 13.50	40, 22.60	88, 25.00	50, 16.00	29, 26.90
II grade obesity	271, 29.90	47, 26.60	47, 13.40	14, 4.50	26, 24.10
III grade obesity	453, 50.10	35, 19.80	26, 7.40	2, 0.60	2, 1.90
WG (*N*, %)					
<Cutoff	109, 12.00	59, 33.30	201, 57.10	254, 81.20	58, 53.70
>Cutoff	796, 88.00	118, 66.70	151, 42.90	59, 18.80	50, 46.30
hs-CRP (*N*, %)					
<1.0 mg/L	126, 13.90	2, 1.10	18, 5.10	170, 54.30	0, 0
1.0–3.0 mg/L	413, 45.60	104, 58.80	277, 78.70	142, 45.40	25, 23.10
>3.0 mg/L	366, 40.40	71, 40.10	57, 16.20	1, 0.30	83, 76.90
PAL (*N*, %)					
Yes	651, 71.90	121, 72.90	204, 58.00	141, 45.00	74, 68.50
No	254, 28.10	56, 31.60	148, 42.00	172, 55.00	34, 31.50
Smoking (*N*, %)					
Yes	654, 71.30	129, 72.90	276, 78.40	259, 82.70	62, 57.40
No	260, 28.70	48, 27.10	76, 21.60	54, 17.30	46, 42.60
Adding sugar (*N*, %)					
Unsweetened	–	83, 46.90	230, 65.30	232, 74.10	64, 59.30
Sweetened	–	94, 53.10	122, 34.70	81, 25.90	44, 40.70

Data are expressed as number (*N*) and percentage (%). BMI, body mass index; WG, waist girth; hs-CRP, high-sensitive C-reactive protein; PAL, physical activity level.

A bivariate proportional odds ratio model was performed to assess the association of coffee consumption (YES/NO) and consumers of sweetened coffee vs. unsweetened coffee with study parameters. Coffee consumption (YES/NO) was associated with all parameters except for age (*p* = 0.170). Similarly, consumers of sweetened coffee vs. unsweetened coffee were associated with all parameters except for age (*p* = 0.929), Xc (*p* = 0.876), and ASMM (*p* = 0.487) (Table 7).

**Table 7:** Bivariate OR model to assess the association of coffee consumption (YES/NO) and consumers of sweetened coffee vs. unsweetened coffee with study parameters.

Parameters	Coffee consumption (YES/NO)				Consumers of sweetened coffee vs. unsweetened coffee			
	OR	*p-value	95% CI	*R* ^2^	OR	*p-value	95% CI	*R*^2^
Age (years)	0.99	0.170	0.989–1.00	0.001	0.99	0.929	0.987–1.012	0.001
BMI (kg/m^2^)	0.80	**<0.001**	0.781–0.813	0.379	1.33	**<0.001**	1.281–1.379	0.346
WG (cm)	0.95	**<0.001**	0.947–0.957	0.223	1.06	**<0.001**	1.051–1.071	0.210
Rz (Ohm)	0.99	**<0.001**	0.991–0.994	0.087	1.01	**<0.001**	1.004–1.008	0.048
Xc (Ohm)	1.02	**<0.001**	1.001–1.028	0.008	0.99	0.876	0.985–1.013	0.001
PhA (°)	5.15	**<0.001**	4.365–6.065	0.257	0.25	**<0.001**	0.194–0.321	0.144
SPA (°)	2.15	**<0.001**	1.941–2.379	0.146	0.60	**<0.001**	0.535–0.683	0.078
SMM (kg)	1.14	**<0.001**	1.123–1.163	0.139	0.94	**<0.001**	0.916–0.956	0.042
ASMM (kg)	1.03	**0.005**	1.008–1.048	0.004	1.01	0.487	0.984–1.035	0.001
hs-CRP (mg/L)	0.81	**<0.001**	0.774–0.850	0.052	1.71	**<0.001**	1.540–1.905	0.129

A *p*-value in bold type denotes a significant difference (*p* < 0.05). OR, odds ratio; IC, interval confidence; BMI, body mass index; WG, waist girth; Rz, resistance; Xc, reactance; PhA, phase angle; SPA, standardised phase angle; SMM, skeletal muscle mass; ASMM, appendicular skeletal muscle mass; hs-CRP, high-sensitive C-reactive protein.

## 4 Discussion

In this cross-sectional study using the 7-day food record, we observed that more than 50% of participants consumed coffee, with 2 cups *per* day being most common, and sweetened coffee preferred over unsweetened. Notably, anthropometric measurements, BC parameters, and inflammatory biomarkers were more favourable among coffee consumers, particularly those drinking 3 cups a day unsweetened. In light of these observations, two key aspects emerge: the positive role of coffee intake and the negative role of excessive and/or constant sugar consumption. These findings suggest that moderate, unsweetened coffee consumption could be considered as part of practical dietary recommendations aimed at improving BC, reducing low-grade inflammation, and potentially preventing metabolic disorders such as obesity, sarcopenia, and IR.

Moreover, coffee consumers, especially those drinking unsweetened coffee, had lower BMI and WG values than their counterparts. Notably, these two parameters showed a J-shape trend with respect to the number of cups of coffee consumed daily, with a minimum peak corresponding to 3 cups *per* day. These results align with previous studies reporting the influence of coffee on BW control and, consequently, on changes in BMI and WG (2, 39, 40), thereby suggesting a potential anti-obesity effect of the beverage, translated into reduced FM via a number of well-established mechanisms of action attributable to the bioactive substances present in the beverage, primarily caffeine (39, 41). In practical terms, such evidence supports the inclusion of moderate coffee consumption within balanced dietary patterns, especially when aiming to prevent central adiposity.

In addition, the effects of regular coffee consumption on improving BC are not only related to a reduction in FM but also to an improvement in the muscle component, exerting a protective effect against the risk of sarcopenia (14–16, 42, 43). The mechanisms for this effect are varied, and one possible mechanism is the regulation of the TGF-*β*/myostatin-Akt-mTORC1 pathway (44). Studies in mouse models have demonstrated further mechanisms, such as a downregulation of myostatin and the stimulation of DNA synthesis in satellite cells (44), resulting in an overall increase in muscle mass (44, 45). However, it is not yet clear what the main component(s) responsible is/are, although caffeine is ruled out, given its effect of attenuating the Akt pathway (44). Consistent with this evidence, our results demonstrate that SMM and ASMM were greater in regular coffee consumers than in non-consumers and increased as the number of cups of coffee consumed increased. This reinforces the potential role of moderate coffee consumption in strategies for maintaining muscle mass during ageing, which could be integrated into nutritional counselling for sarcopenia prevention.

The study of SMM is of relevant importance. SMM, indeed, is currently considered to be a metabolically active endocrine and paracrine organ that is capable of communicating with different organs and systems via specific proteins called myokines (46, 47). Its metabolic involvement lies essentially in the establishment of an IR condition as a result of SMM loss, which, in turn, is responsible for alterations in glucose and lipid homeostasis, as well as being itself implicated in muscle catabolism phenomena (47, 48). Furthermore, a similar bidirectional relationship is also observed in relation to the inflammatory state (47). Whilst levels of markers of inflammation (including CRP and interleukin (IL)-6) have been reported to be inversely associated with ASMM (49), tumour necrosis factor (TNF)-*α* has been shown to contribute to muscle catabolism via activation of NF-κB (50).

Interestingly, in addition to the results on SMM and ASMM, in our study, we also observed lower hs-CRP levels in regular coffee consumers than in non-consumers, and these levels tended to decrease as the number of cups consumed increased, from 0 to 3 *per* day. Taken together with the abovementioned evidence, we can speculate a possible indirect anti-inflammatory effect of coffee exerted via its direct anti-catabolic effect on SMM. At the same time, we infer the usefulness of the BIA as a useful and validated tool (51–53) for studying SMM and ASMM and their changes over time, making it possible to obtain important translational information about the subject’s metabolic and inflammatory status.

In addition to these potential indirect mechanisms, it is well known that coffee, or its bioactive components, exerts a marked antioxidant and anti-inflammatory effect (1). Among the main mechanisms are (i) regulation of reactive oxygen species production, reduction of pro-inflammatory cytokine levels, and increased Nrf-2 expression (mainly due to caffeine) and (ii) free radical scavenging action, downregulation of pro-inflammatory cytokine gene expression, and regulation of NF-kB activation (mainly due to chlorogenic acid) (1). These findings are corroborated by animal model studies demonstrating the effect of coffee consumption on reducing levels of inflammatory markers such as IL-1α, IL-6, and TNFα (45).

Of particular note, we observed for the first time that, among coffee consumers, the highest SMM and ASMM and the lowest hs-CRP values were found among those who consumed unsweetened coffee. Although this latter result on inflammation was expected, given the effect of excessive and/or regular sugar consumption on promoting the inflammatory state (19), the observation regarding the impact of adding sugar on the muscle component is of interest.

Previous studies have investigated this, showing that chronic sugar intake from various food sources was associated with lower muscle mass index values in adolescents (17) and reduced hand grip strength in middle-aged and elderly subjects (18). According to the authors, underlying these observations are direct actions of sugar on muscle cells, interfering with telomere length and autophagy, apoptosis, and senescence mechanisms (54, 55). Similarly, the effect of sugar on the stimulation of oxidative and inflammatory pathways has been reported (56, 57), thereby feeding the vicious circle described above that sees the production of proinflammatory cytokines as a negative stimulus on the muscle component (58, 59). Conversely, in gastrocnemius samples from an aged rat model, the chronic administration of polyphenols resulted in increased antioxidant capacity and activity of antioxidant enzymes (i.e., catalase, glutathione peroxidase, and glutathione reductase), reduction of lipid (malondialdehyde) and protein (N-tyrosine) oxidation markers, upregulation of antioxidant genes (catalase and glutathione peroxidase), and downregulation of pro-inflammatory genes (IL6). These biochemical results were accompanied by an improvement in motor performance and coordination (60), suggesting the protective effect of polyphenols in counteracting the negative effects of ageing on muscles.

Other observations in animal models, however, also suggest a metabolic effect, showing that chronic sugar intake alters the insulin and AMPK pathways in the muscle (gastrocnemius), resulting in a reduction in the expression and membrane translocation of the GLUT-4 and GLUT-5 transporters (20). The IR induced by chronic sugar intake represents a further key aspect, considering that (i) the consequent stimulation of lipolysis results in an increased release of free fatty acids that inhibit the GH/IGF-1 pathway, with a negative effect on muscle regeneration (48) and (ii) the altered suppression of gluconeogenesis induced by compensatory hyperinsulinemia promotes proteolysis and inhibits protein synthesis, causing a loss of SMM (61, 62). These mechanisms make it possible to justify our observation of reduced SMM and ASMM values among sweetened coffee consumers and, once again, allow for a translational interpretation of changes in these BIA parameters for monitoring nutritional status.

In this context, our results regarding the anti-inflammatory effect of moderate consumption of unsweetened coffee are also supported by observations of other BIA parameters, including PhA and SPA. From a hydro-electrolyte balance point of view, the inflammatory state is characterised by an expansion of extracellular fluids (63). This phenomenon is reflected at the BIA level with an increase in extracellular water levels and a reduction in intracellular water and, consequently, a reduction in PhA values (64), which is therefore considered a surrogate marker of inflammation (21). Our results show that the highest PhA values were found among coffee consumers and among those who consumed unsweetened coffee, thus varying in parallel with hs-CRP levels. The same trend was followed by SPA values, a more accurate parameter which, by normalising PhA by sex and age, makes it possible to assess the average PhA values of a specific category of subjects within a normal population (31, 32). Overall, therefore, our results confirm the effect of moderate coffee consumption (preferably unsweetened) in modulating the inflammatory state. Furthermore, the beneficial effects of moderate coffee consumption (2–3 cups *per* day) that we have observed are further supported by previous studies demonstrating an increase in inflammatory parameters with excessive coffee consumption (≥4 cups *per* day) (37, 65–67), suggesting a potential hormetic effect of the beverage’s bioactive components.

In summary, moderate, unsweetened coffee consumption appears associated with favourable BC and inflammatory profiles, with implications for sarcopenia and metabolic health. However, as this is an observational study, causality cannot be inferred.

### 4.1 Limitations and strengths

We are aware of some limitations in the study. First, our study’s cross-sectional design does not allow us to determine cause-and-effect relationships between coffee consumption and the clinical parameters evaluated. In addition, it was not possible to establish any linear dose–response effect. Second, because we did not include decaffeinated coffee consumers in the statistical analysis, we could not determine the relative contribution of caffeine or polyphenols to our results. Third, although the sample size is large, the distribution between sexes is unequal. Fourth, we do not have information on either the coffee variety (e.g., Arabica, Robusta, Liberica, and Excelsa) or the preparation method (e.g., coffee from the café/commercial preparation or home preparation). This may vary the composition of the beverage in terms of bioactive compounds with respect to both the blend and the extraction method. Fifth, the lack of data on daily caloric intake prevents us from establishing whether differences in BC and inflammation are causally related or independently associated with coffee consumption. Future studies along these lines may help to understand whether coffee intake may influence daily caloric intake. However, one important strength of our study is that we excluded occasional coffee consumers from the statistical analysis. This reduced possible confounding variables. Additional strengths include the use of the 7-day food diary, which allowed for better characterisation of the coffee consumption habits, in terms of daily servings and sugar addition. This is important for understanding the real impact of coffee on BC and inflammation, since as coffee consumption increases, so does sugar intake, which negatively affects caloric intake and metabolic balance. Notably, this method is considered the “gold standard” in validation studies of different types of self-administered food frequency questionnaires. Finally, because coffee preparation methods vary widely, an additional strength of this study is the recruitment of subjects from the same geographical origin who consumed Italian-style coffee, thereby reducing the variability of the results.

## 5 Conclusion

Regular coffee consumption appears to have a positive impact on BC and inflammatory status. Compared to non-coffee consumers, regular coffee consumers presented (i) lower BMI, WG, and hs-CRP levels and (ii) higher SMM, ASMM, PhA, and SPA assessed by BIA. These effects also seem to depend on the habit of consuming sweetened coffee and the number of cups consumed daily. Chronic low-grade inflammation is recognised as a key pathogenic factor in the development of sarcopenic obesity, where the concomitant loss of muscle mass and gain of FM create a self-perpetuating cycle of metabolic deterioration. In this context, the observed association between regular coffee consumption and lower hs-CRP levels supports the hypothesis that coffee, due to its anti-inflammatory properties, may help modulate inflammatory pathways and thus reduce the risk of sarcopenic obesity. Importantly, groups at increased risk of sarcopenic obesity—including older adults, individuals with sedentary lifestyles, and patients with chronic diseases—may particularly benefit from moderate, unsweetened coffee consumption as part of nutritional strategies aimed at improving BC and preserving muscle function. Incorporating coffee into a balanced diet, alongside regular physical activity, could therefore represent a simple, practical approach to support muscle health and reduce inflammation in these vulnerable populations. The results of this study, therefore, suggest that, although regular coffee consumption (in the context of a balanced diet and an active lifestyle) can be considered beneficial for maintaining good health, it is advisable both to avoid added sugar and to pay attention to the amount consumed throughout the day, a maximum of 3 cups, in order to enhance its beneficial effects. Clearly, these observations are to be considered general and not universally applicable. In fact, even the indications for coffee intake must be tailored to the habits, preferences, and needs of the individual, taking into account any absolute contraindications to its consumption, such as specific diagnoses or drug therapies. However, these findings may help guide dietary recommendations aimed at promoting muscle maintenance in specific conditions, such as weight loss programmes, to reduce the risk for sarcopenic obesity.

## Data Availability

The datasets analyzed during the current study are available from the corresponding author on reasonable request.
